# Molecular Flexibility and Bend in Semi‐Rigid Liquid Crystals: Implications for the Heliconical Nematic Ground State

**DOI:** 10.1002/chem.201903677

**Published:** 2019-10-17

**Authors:** Richard J. Mandle, John W. Goodby

**Affiliations:** ^1^ Department of Chemistry University of York York YO10 5DD UK

**Keywords:** conformer libraries, liquid crystals, NMR spectroscopy, soft matter

## Abstract

The N_TB_ phase phases possess a local helical structure with a pitch length of a few nanometers and is typically exhibited by materials consisting of two rigid mesogenic units linked by a flexible oligomethylene spacer of odd parity, giving a bent shape. We report the synthesis and characterisation of two novel dimeric liquid crystals, and perform a computational study on 10 cyanobiphenyl dimers with varying linking groups, generating a large library of conformers for each compound; this allows us to present molecular bend angles as probability weighted averages of many conformers, rather than use a single conformer. We validate conformer libraries by comparison of interproton distances with those obtained from solution‐based 1D ^1^H NOESY NMR, finding good agreement between experiment and computational work. Conversely, we find that using any single conformer fails to reproduce experimental interproton distances. We find the use of a single conformer significantly overestimates the molecular bend angle while also ignoring flexibility; in addition, we show that the average bend angle and flexibility are both linked to the relative stability of the N_TB_ phase.

## Graphical abstract

**Bend it like Mandle**! Conformational analysis of liquid crystalline dimers, validated by comparison of computed and experimental ^1^H NOESY NMR data is used to analyse average molecular shape, which is demonstrated to underpin the formation of the liquid crystalline heliconical nematic N_TB_ phase.

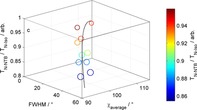

## Introduction

Liquid crystals (LC) are a collection of states of matter with some degree of positional or orientational organisation and are widely employed as functional materials.^1^, ^2^, ^3^ Different LC mesophase are principally characterised by their degree of orientational and/or positional order; for example, the nematic LC phase possesses long range orientational order with short range positional order, whereas lamellar phases also exhibit positional order in one dimension. The twist‐bend modulated nematic phase (TB), the average orientation of the nematic phase rotates over a few nanometers giving a helical structure with a remarkably short pitch length.^4^, ^5^, ^6^, ^7^, ^8^, ^9^ The N_TB_ phase is therefore chiral despite being typically formed by achiral molecules, although a handful of chiral materials are also known to exhibit this phase.^10^, ^11^, ^12^ Although this phase is principally exhibited by liquid‐crystalline dimers, in which two rigid sections are adjoined by a (semi‐) flexible spacer,^13^, ^14^ it has also been observed in semi‐rigid bent‐core materials,^15^ liquid crystalline *n*‐mers^16^, ^17^, ^18^, ^19^ and polymers.^20^ Experimental results suggest it is primarily molecular shape^14^, ^21^, ^22^ and the gross bend‐angle which appear to dictate the incidence of this phase,^14^, ^23^, ^24^ supporting the findings of earlier theoretical treatments.^24^, ^26^

Arakawa et al. recently reported several cyanobiphenyl dimers containing methylene spacers with various combinations of chalcogen linkages (ether, thioether, selenoether).^27^, ^28^ Arakawa et al. found that the all *trans* form of the *bis* selenoether is the most bent (90°), followed by the *bis* thioether (109°), the thioether/ether (126°) and finally the *bis* ether (144°). The relationship between T_NTB−N_ and T_N−Iso_ and the bend angle of the all *trans* conformer is superficially similar to that reported by the York group previously.^24^

**Figure 1:**
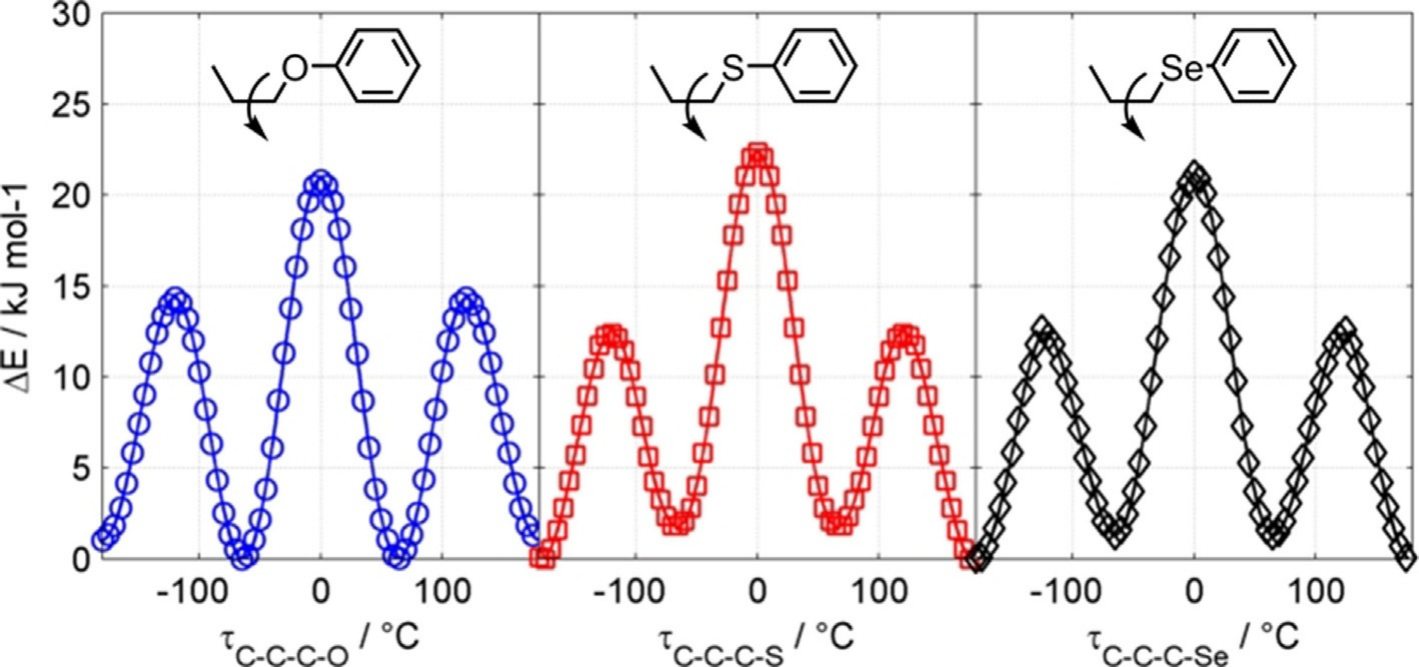
Plot of energy as a function of dihedral angle for propoxybenzene (blue, left), phenyl(propyl)sulfane (red, centre), and phenyl(propyl)selane (black, right), calculated by performing fully relaxed scans using the wB97XD hybrid functional^30^ and the aug‐cc‐pVTZ basis set.^31^ Solid lines are spline fits to the computational data, and are presented as a guide to the eye.

The use of only the all *trans* conformer for calculating bend neglects contributions from conformers containing one or more dihedrals in a *gauche* conformation; for simple molecules the difference in energy between *trans* and *gauche* states can be sufficiently low that both are significantly populated, as shown for propoxybenzene (and its thio‐ and seleno‐ analogues) in Figure 1. If we now consider liquid crystalline dimers incorporating long flexible spacers, the contributions of *gauche* conformations to the average molecular bend may be too significant to be ignored.^23^, ^29^ Herein we perform a computational study of the conformational landscape of these materials from our earlier work, as well as the thio‐ and seleno‐ analogues reported recently by Arakawa et al. as well as two novel materials.

## Experimental Section

**Scheme 1:**
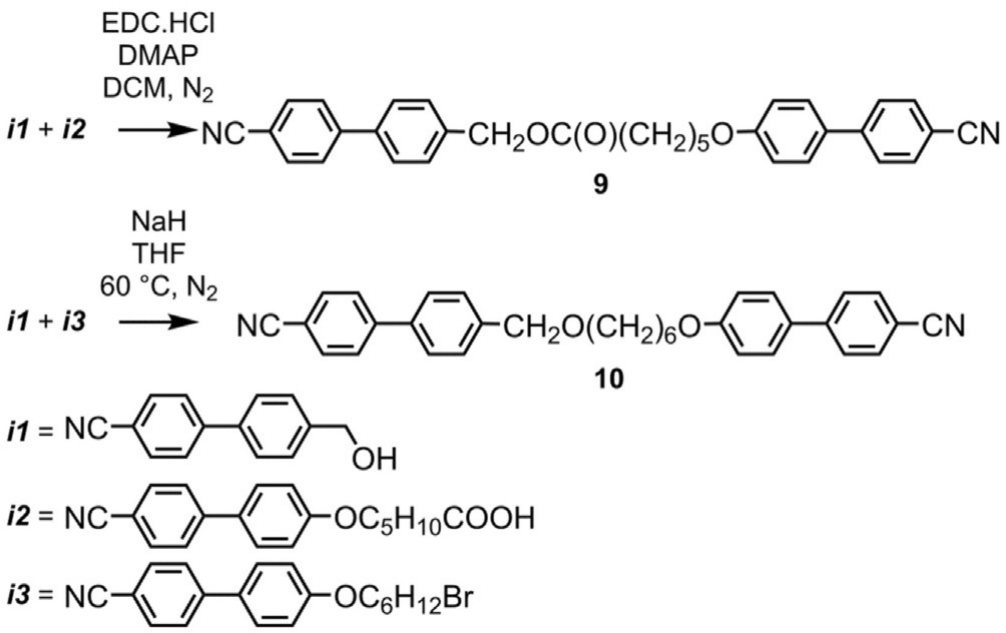


Compound **1** has been extensively studied,^32^ compounds **2**–**5** were reported in ref. 24, compounds **6**, **7** and **8** were reported by Arakawa et al. in ref. 27 and 28. Compounds **9** and **10** were synthesised as part of this work according to Scheme 1. Steglich esterification of ***i1*** with ***i2*** afforded compound **9**, whereas compound **10** was prepared by Williamson etherification of ***i1*** with ***i3***. The synthetic intermediates 4‐(4‐cyanophenyl)benzyl alcohol (***i1***),^33^ 6‐((4′‐cyano‐[1,1′‐biphenyl]‐4‐yl)oxy)hexanoic acid (***i2***),^34^ 4′‐((6‐bromohexyl)oxy)‐[1,1′‐biphenyl]‐4‐carbonitrile (***i3***)^35^ were synthesised according to literature precedent. Full details are given in the Supporting Information.

Polarised optical microscopy (POM) was performed on a Zeiss Axioskop 40Pol microscope using a Mettler FP82HT hotstage controlled by a Mettler FP90 central processor. Differential scanning calorimetry (DSC) was performed on a Mettler DSC822e calibrated before use against indium and zinc standards under an atmosphere of dry nitrogen. DSC thermograms were processed in Matlab. For compounds **9** and **10** phase assignment was made by POM, while transition temperatures and associated enthalpies were determined by DSC. The conditions used for recording one dimensional ^1^H NOESY NMR are reported in ref. 24. Computational chemistry was performed using Gaussian 16 revision A.03 suite of programmes.^36^

## Results

We selected 10 dimeric compounds with 4‐cyanobiphenyl mesogenic units and a spacer containing a longest linear sequence of nine non‐hydrogen atoms, that is, methylene or equivalent. Transition temperatures and molecular structures are given in Table 1.

**Table 1:** Transition temperatures and molecular structures of 10 dimeric compounds with 4‐cyanobiphenyl mesogenic units and a spacer containing a long linear sequence of nine non‐hydrogen atoms, that is, methylene or equivalent.^[a]^

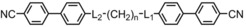							
No.	L_1_	*n*	L_2_	*T*_MP_	*T*_NTB−N_	*T*_N−Iso_	δ*T*
1	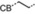	5	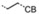	83.3	105.4	121.5	0.96
2	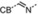	5	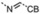	140.8	114.7	138.7	0.94
3	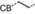	5	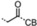	127.8	128.1	153.9	0.94
4	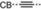	5	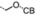	137.1	102.0	153.6	0.88
5	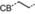	5	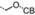	110.6	109.9	153.3	0.90
6	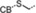	5	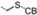	15.9^[b]^	88.3	115.2	0.93
7	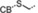	5	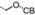	55.0	95.9	146.7	0.88
8	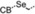	5	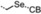	80.8–101.4	43.1	71.9	0.91
9	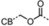	4	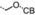	95.1 [24.8]	39.8 [<0.1]	91.3 [0.9]	0.86
10	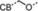	5	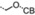	122.4 [12.4]	71.3 [<0.1]	129.9 [0.5]	0.85

[a] Transition temperatures [°C] of compounds **1**–**5** obtained from DSC at a heat/cool rate of 10 ° min^−1^.^24^ Values for **6** and **7** were taken from ref. 27, values for **8** were taken from ref. 28. Compounds **9** and **10** were studied as part of this work; transition temperatures and associated enthalpies were obtained from DSC at a heat/cool rate of 10° min^−1^. The scaled transition temperature (δ*T*) is defined here as *T*_NT−N_/*T*_N−Iso_. [b] Glass to N_TB_ transition, * CB=4‐cyanobiphenyl. Associated enthalpies of transition (kJ mol^−1^) are given in square parenthesis for novel compounds (**9** and **10**).

Compounds **1**–**8** are known to exhibit nematic and N_TB_ phases; the novel materials **9** and **10** both exhibit nematic and N_TB_ phases, with phase identification made by POM and DSC. Each material in Table 1 has a nonamethylene or equivalent central alkyl spacer, that is to say, each compound has a longest linear sequence of nine non‐hydrogen atoms; we chose to exclude materials which possess a nonamethylene equivalent spacer but do not exhibit the N_TB_ phase (e.g. CBO7OCB, CBCC≡C5C≡CCB).^23^

**Figure 2:**
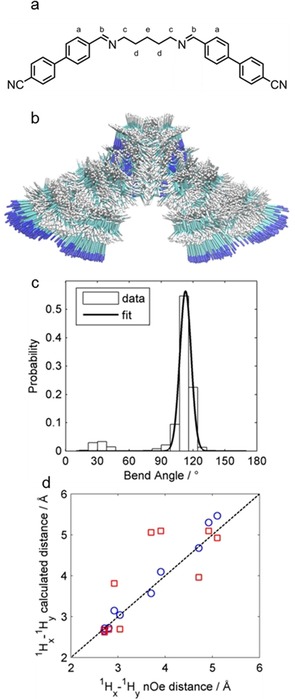
(a) Molecular structure of compound **2**, with proton environments used in NOE distance measurements labelled. (b) Overlaid image of all populated conformers (Δ*E* ≤20 kJ mol^−1^ at 298 K) of compound **2** obtained as described in the text. (c) Histogram plot of calculated bend angle probabilities of compound **2**, with a single Gaussian fit to the major peak at ≈110°. (d) Plot of Interproton distances (H_*x*_−H_*y*_) obtained from calculations versus those from ^1^H NOE NMR: red squares show calculated interproton distances from a single conformer (the all *trans* global minimum) whereas blue circles show interproton distances which are a probability weighted average of many conformations.

Next we generated a library of conformers for each compound according to the RIS model, as described by Archbold et al.^23^ We performed relaxed scans about each dihedral in the central spacer of **1**–**10**, allowing each dihedral to undergo threefold rotation. This afforded a library of 3^n^ conformers (for a material with *n* alkane dihedrals) for each material which was subsequently pruned by discarding conformers whose energy was greater than the global energy minimum by 20 kJ mol^−1^ and/or had one or more pairs of atoms closer than a cut‐off distance of 0.8× the sum of their van der Waals radii. From the Cartesian coordinates of the 4 and 4′ carbon atoms of each biphenyl we calculated the angle between the two mesogenic units for all remaining conformers. We use the energy of each conformer to give a Boltzmann population, allowing us to present bend angles which are the probability weighted average of many conformers (Figure 2). This better reflects the inherent flexibility of these compounds than the use of a single conformer, but, neglects the influence of the local nematic or twist‐bend nematic director upon conformer probability. When calculating Boltzmann distributions we assume a temperature of 298 K.

We considered that for a flexible molecule incorporating an oligomethylene portion, such as an LC dimer, the validity of a conformer library is principally determined by the probability assigned to a given conformer. We therefore sought an experimental verification that the conformer libraries we generated were usable. Solution based one dimensional proton nuclear Overhauser effect (1D ^1^H NOE) NMR allows us to relate the intensity of observed NOE enhancements to calculate interproton distances by using a known interproton distance as a reference. We selected compound **2** for study by 1D ^1^H NOE NMR as it has well separated signals (permitting selective saturation of given resonances), a relatively small number of populated conformers (due to the *bis* imino linkers) and only four distinct proton resonances associated with its central spacer (thereby reducing the number of NOE experiments required). We use the *ortho* CH−CH distance in the aryl rings as a standard distance, from this we calculate all interproton distances of interest based only on measured NOE enhancements adjusted for the number of chemically equivalent spins in the two proton environments that give rise to each signal. We then compared these to the interproton distances obtained by taking a probability weighted average from each conformer (Table 2). There is good agreement between interproton distances from 1D ^1^H NOE NMR and those from taking a probability weighted average of many conformers (average difference ≤5 %). For larger interproton distances (>5 Å, for example, H_a_–H_e_) the NOE method gives smaller values than those obtained computationally which we consider to be due to the low intensity of the NOE signal and thus low S/N ratio. We find that using interproton distances from any single conformer—even the global energy minimum—fails to reproduce the expected NOE enhancements for non‐adjacent proton environments, giving an average discrepancy of >15 % (Figure 2 d). When calculating geometrical parameters, be they internuclear distances or average bend‐angles, this highlights the importance of accounting for contributions from all populated conformers.

**Table 2:** Interproton distances obtained by taking a probability weighted average from each conformer.^[a]^

	*H_a_*	*H_b_*	*H_c_*	*H_d_*	*H_e_*
*H_a_*	–	3.0 (3.0)	4.7 (4.7)	5.1 (5.5)	4.9 (5.3)
*H_b_*	3.0 (3.0)	–	2.9 (3.2)	3.7 (3.6)	3.9 (4.1)
*H_c_*	4.7 (4.7)	2.9 (3.2)	–	2.7 (2.6)	2.7 (2.7)
*H_d_*	5.1 (5.5)	3.7 (3.6)	2.7 (2.6)	–	2.7 (2.7)
*H_e_*	4.9 (5.3)	3.9 (4.1)	2.7 (2.7)	2.9 (2.7)	–

[a] Interproton distances [Å] of compound **2** as measured from 1D NOE NMR intensities (500 MHz, CDCl_3_) and, in parenthesis, interproton distances as a probability weighted average of many conformers.

**Figure 3:**
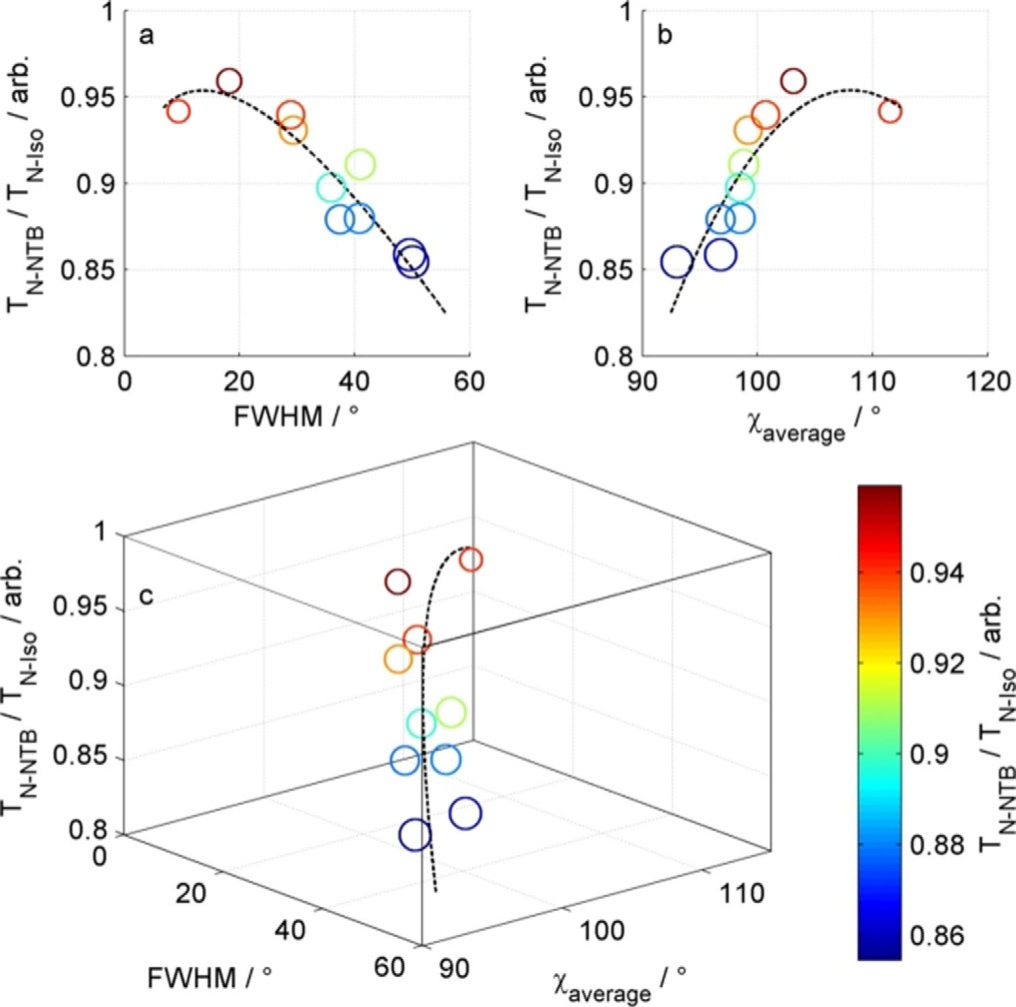
Plots of the scaled transition temperature (δ*T*, *T*_NTB−N_/*T*_N−Iso_) as a function of the FWHM (a, *Y*–*Z* plane of Figure 3c) and as a function of the average bend angle (b, *X*–*Z* plane of Figure 3c) for **1**–**10**. (c) Plot of the scaled transition temperature (δ*T*,*T*_NTB−N_/*T*_N−Iso_) versus FWHM [°] versus average bend angle [°]. The dashed line is a line of best fit to the data and is presented as a guide to the eye. The size of each data point is inversely proportional to the FWHM, and are coloured according to the *T*_NTB−N_/*T*_N−Iso_ as shown by the scale bar on the right.

As noted by Archbold et al.,^23^ two materials can have comparable average bend angles but radically different conformer distributions due to significant populations of hairpin or linear conformer or simply a broad range of bend angles. To quantify this we fitted the histogram with a Gaussian centred on the probability weighted average bend angle (Figure 2 c). To remove the effect of histogram bin size on the FWHM we vary the number of bins from 180 to 20, and take an average. We use this average FWHM of the Gaussian fit as a measure of the breadth of the conformer distribution (i.e. the range of populated bend angles) and so enabling comparison between materials. Data is presented numerically in Table 3 and graphically in Figure 3.

**Table 3:** Average bend angles, bend angles of the global energy minimum conformer, FWHM values, and scaled transition temperatures of compounds **1**–**10**.^[a]^

No.	δ*T*	$\chi_{min}$	$\chi_{ave}$	$\chi_{FWHM}$
**1**	0.96	111.9	103.1	16.9
**2**	0.94	125.1	111.5	9.4
**3**	0.94	108.5	98.5	40.8
**4**	0.88	126.7	98.5	35.9
**5**	0.90	118.2	100.7	30.0
**6**	0.93	120.2	99.2	22.0
**7**	0.88	130.2	96.8	37.4
**8**	0.91	114.3	98.8	41.2
**9**	0.86	109.6	93.0	50.1
**10**	0.85	131.6	96.8	49.5

[a] Scaled transition temperatures (δ*T*, *T*_NTB−N_/*T*_N−Iso_), bending angle for the global energy minimum conformer ($\chi_{min}$ ), probability weighted average bending angle ($\chi_{ave}$ ), full‐width at half maximum ($\chi_{FWHM}$ ) determined as described in the text.

Table 3 presents average bend angles, bend angles of the global energy minimum conformer, FWHM values, and scaled transition temperatures of compounds **1**–**10**. This is of interest as we previously suggested that a tight distribution of conformers centred about a favourable bend angle can lead to direct isotropic to N_TB_ transitions, that is, *T*_NTB−N_/*T*_N−Iso_ of ≥1. If we invert this argument then it is expected that as the FWHM is broadened T_NTB−N_/T_N−Iso_ would decrease, with the nematic phase range expanding until the material no longer exhibits the twist‐bend phase. This is observed experimentally (Figure 3 a) as materials which show larger FWHM values exhibit smaller scaled N_TB_–N transition temperatures (Figure 3 a). The scaled N_TB−N_ transition temperature also displays a dependence on the average bend angle (Figure 3 b), increasing as the average molecular bend increases, reaching a maximum value in the region 100–110° and then decreasing again as the angle increases further.

In reality, the relationship between the scaled transition temperature and either the FWHM or average bend angle cannot be taken in isolation. A favourable FWHM and unfavourable bend angle (or vice versa) would not be expected to generate the N_TB_ phase: consider the large number of rigid bent‐core liquid crystals which do not exhibit twist‐bend nematic phases,^37^ although these may be hidden by the “smectic blanket”.^38^ In this work, the materials studied feature a methylene spacer with varying linking group; it is therefore to be expected that as linkers are used which introduce greater flexibility (increased FWHM) the average bend angle decreases further away from the tetrahedral angle imposed by the methylene spacer. A 3D plot of scaled transition temperature versus FWHM versus average bend shows how these three properties are related (Figure 3 c). Clearly, both average bend and flexibility play a part in dictating the scaled transition temperature and this can explain the previously unexpectedly low values of δ*T* of materials such as CBS3SCB^27^, ^39^ and CBO5OCB,^24^, ^40^ both of which satisfy the angular dependency of the N_TB_ phase but are highly flexible.

Further studies into the relationship between molecular bend and the incidence of the N_TB_ phase will be expected to be complicated by the difficulty in preparing spacers which give bend angles larger than those presented here without introducing flexibility (i.e. a large FWHM), which will suppress the formation of the N_TB_ phase. One possible route would be liquid crystalline n‐mers incorporating mixed odd‐ and even‐ parity spacers; the average of the two bending angles would be expected to be larger than can be achieved by an odd‐parity methylene spacer.

## Addendum

Although Imrie et al. independently reported the synthesis and transition temperatures of these two materials,^39^ in this work we chose to use transition temperatures for compounds **6** and **7** reported by Arakawa et al.^27^ because the clearing points are marginally higher. For example, *T*_NTB−N_ of **7** reported by Imrie et al. is 8 °C lower than that reported by Arakawa et al. Nevertheless, the effect on the scaled transition temperature is actually minimal (using the values reported by Imrie et al. we obtain *T*_NTB−N_/*T*_N−Iso_ values of 0.92 and 0.86 for **6** and **7**, respectively) and does not impact the conclusions drawn from this work.

## Conclusions

The conformational space of 10 cyanobiphenyl based dimers—with varying linking group and a spacer made up of nine non‐hydrogen atoms—has been explored. Each material exhibited exhibits nematic (N) and twist‐bend nematic (N_TB_) mesophases. We calculate the average molecular bend and flexibility (FWHM) for each material. The probability weighted average interproton distances obtained computationally are in good agreement with those obtained by solution based 1D ^1^H NOE NMR. The average bend angle and FWHM are shown to be correlated with the scaled transition temperature (*T*_NTB−N_/*T*_N−Iso_). The present results support the previous view that a “tight” conformer distribution centred in the range 100–100° underpins the formation of the N_TB_ phase. This work also shows that for flexible systems such as LC dimers the use of a single conformer to determine average bend is unsatisfactory; others are encouraged to adopt the use of conformer libraries when discussing geometric molecular properties of these systems.

## Conflict of interest

The authors declare no conflict of interest.

## Supporting information

As a service to our authors and readers, this journal provides supporting information supplied by the authors. Such materials are peer reviewed and may be re‐organized for online delivery, but are not copy‐edited or typeset. Technical support issues arising from supporting information (other than missing files) should be addressed to the authors.

Supplementary
