# Supplementary material for: Molecular Flexibility and Bend in Semi‐Rigid Liquid Crystals: Implications for the Heliconical Nematic Ground State
Source: Chemistry. 2019 Oct 17;25(63):14454–9. doi: 10.1002/chem.201903677 (PMC6899767; doi:10.1002/chem.201903677)
Supplement: Supplementary file 1 — Supplementary [file CHEM-25-14454-s001.pdf]

CHEMISTRY
A European Journal

Supporting Information

Molecular Flexibility and Bend in Semi-Rigid Liquid Crystals: Implications for the Heliconical Nematic Ground State

Richard J. Mandle* and John W. Goodby[a]

chem_201903677_sm_miscellaneous_information.pdf

1. Methods

1.1. General Techniques

Miscellaneous solvents were purchased from Fisher Scientific dried by sequential percolation through columns of activated alumina and copper Q5 catalyst prior to use. Unless otherwise noted, chemical reagents were purchased from commercial suppliers and used without further purification. Intermediates ***i1***, ***i2*** and ***i3*** were prepared according to literature precedent. [1-3]

Reactions were monitored by thin layer chromatography (TLC) using an appropriate solvent system. Silica coated aluminium TLC plates used were purchased from Merck (Kieselgel 60 F-254) and visualised using UV light at wavelengths of both 254 nm and 365 nm. Column chromatography was performed using flash grade silica from Fluorochem (40 - 63μm particle size). Yields refer to chromatographically (HPLC) and spectroscopically (^{1}H NMR and ^{13}C {^{1}H} NMR) homogenous material.

1.2. Nuclear Magnetic Resonance

NMR spectra were recorded on a JEOL ECS spectrometer operating at 400 MHz (^{1}H) and 100.5 MHz (^{13}C{^{1}H}) as solutions in deuterated chloroform. Spectra were referenced to the residual protic solvent for ^{1}H (7.26 ppm), ^{13}C{^{1}H} to the resonance of $CDCl_3$ (77.16 ppm).

1.3. Mass Spectrometry

Mass spectra were recorded on a Bruker compact time of flight mass spectrometer with both ESI and APCI sources, and we extend our gratitude to Mr. Karl Heaton of the University of York for obtaining MS data.

1.4. Polarised Optical Microscopy

Polarised optical microscopy was performed on a Zeiss Axioskop 40Pol microscope using a Mettler FP82HT hotstage controlled by a Mettler FP90 central processor. Photomicrographs were captured *via* an InfinityX-21 MP digital camera mounted atop the microscope.

1.5. Differential Scanning Calorimetry.

Differential scanning calorimetry was performed on a Mettler DSC822^e fitted with an autosampler operating with Mettler Star^e software and calibrated before use against an indium standard (onset = 156.55 ± 0.2 °C, ΔH = 28.45 ± 0.40 Jg^{-1}) under an atmosphere of dry nitrogen.

1.6. Computational Chemistry

Quantum chemical calculations were performed using the Gaussian 16 revision A.03 suite of programmes. [4] Conformer libraries were generated as described by Archbold *et al.* [5]

2. Chemical Characterisation

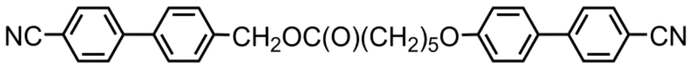

9:

A suspension of ***i1*** (209 mg, 1 mmol), ***i2*** (309 mg, 1 mmol), EDC.HCl (191 mg, 1 mmol) and DMAP (10 mg) in DCM (10 ml) was stirred until complete consumption of ***i1***. The crude reaction mixture was purified by flash chromatography over silica gel with DCM as the eluent. The chromatographed material was recrystalised from ethanol, giving the title compound as white microcrystals.

Yield: 295 mg (59 %)

^{1}H NMR: 1.48-1.56 (2H, m, $-CH_2-C\underline{H}_2-CH_2$), 1.70-1.90 (4H, m, $-CH_2(C\underline{H}_2)-CH_2-$), 2.43 (2H, t, *J* = 7.5 Hz, $Ar-CH_2-OOC-C\underline{H}_2-CH_2-$) 3.98 (2H, t, *J* = 6.4 Hz, $Ar-O-C\underline{H}_2CH_2-$), 5.17 (2H, s, $Ar-C\underline{H}_2-O-CH_2$), 6.97 (2H, ddd, *J* = 1.8 Hz, *J* = 3.1 Hz, *J* = 8.8 Hz, Ar$\underline{H}$), 7.42-7.74 (14H, m, Ar$\underline{H}$)

^{13}C{^{1}H} NMR: 24.75, 25.74, 28.98, 34.25, 65.72, 67.84, 110.17, 111.20, 115.12, 118.94, 119.20, 127.15, 127.52, 127.53, 127.77, 128.42, 128.99, 131.46, 132.73, 136.73, 139.15, 145.14, 145.28, 159.73, 173.45

MS (ESI): 509.2198 (calcd. for $C_{33}H_{30}N_2NaO_2$: 509.2199, M + Na)

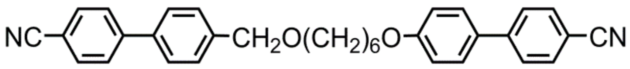

10:

Sodium hydride (60% dispersion in mineral oil, 80 mg, 2 mmol) was added to a stirred solution of ***i1*** (200 mg, 0.96 mmol) in anhydrous THF (10 ml) under an atmosphere of dry nitrogen at 0 °C. ***i3*** (342 mg, 0.96 mmol) was added as one portion, and the solution heated under reflux for 2 h. The suspension was cooled to room temperature; water added (5 ml), and stirring continued for 1 h. The solvents were removed *in vacuo*. The crude residue was suspended in DCM and filtered over a silica plug, eluting with DCM. The crude material was recrystalised from ethanol, giving the title compound as a white solid.

Yield: 310 mg (66 %)

^{1}H NMR: 1.44-1.54 (4H, m, $-CH_2-(C\underline{H}_2)_2-CH_2$), 1.63-1.72 (2H, m, $-CH_2C\underline{H}_2-CH_2-$), 1.77-1.85 (2H, m, $-CH_2C\underline{H}_2-CH_2-$), 3.52 (2H, t, *J* = 6.5 Hz, $Ar-CH_2-O-C\underline{H}_2-CH_2-$), 3.99 (2H, t, *J* = 6.5 Hz, $Ar-O-C\underline{H}_2-CH_2-$), 4.55 (2H, s, $Ar-C\underline{H}_2-O-CH_2-$), 6.97 (2H, ddd, *J* = 2.0 Hz, *J* = 3.1 Hz, *J* = 8.7 Hz, Ar$\underline{H}$), 7.42-7.46 (2H, m, Ar$\underline{H}$), 7.50 (2H, ddd, *J* = 2.0 Hz, *J* = 3.1 Hz, *J* = 8.7 Hz, Ar$\underline{H}$), 7.55 (2H, ddd, *J* = 1.6 Hz, *J* = 1.8 Hz, *J* = 8.2 Hz, Ar$\underline{H}$), 7.60-7.72 (8H, m, Ar$\underline{H}$).

^{13}C{^{1}H} NMR: 26.01, 26.10, 29.27, 29.80, 68.10, 70.65, 72.50, 110.15, 115.14, 119.02, 119.20, 127.15, 127.33, 127.72, 128.36, 128.41, 131.40, 132.66, 132.69, 138.45, 139.43, 145.32, 145.46, 159.82

MS (ESI): 523.1986 (calcd. for $C_{33}H_{28}N_2NaO_3$: 523.1992, M + Na)

3. Supplementary Figures

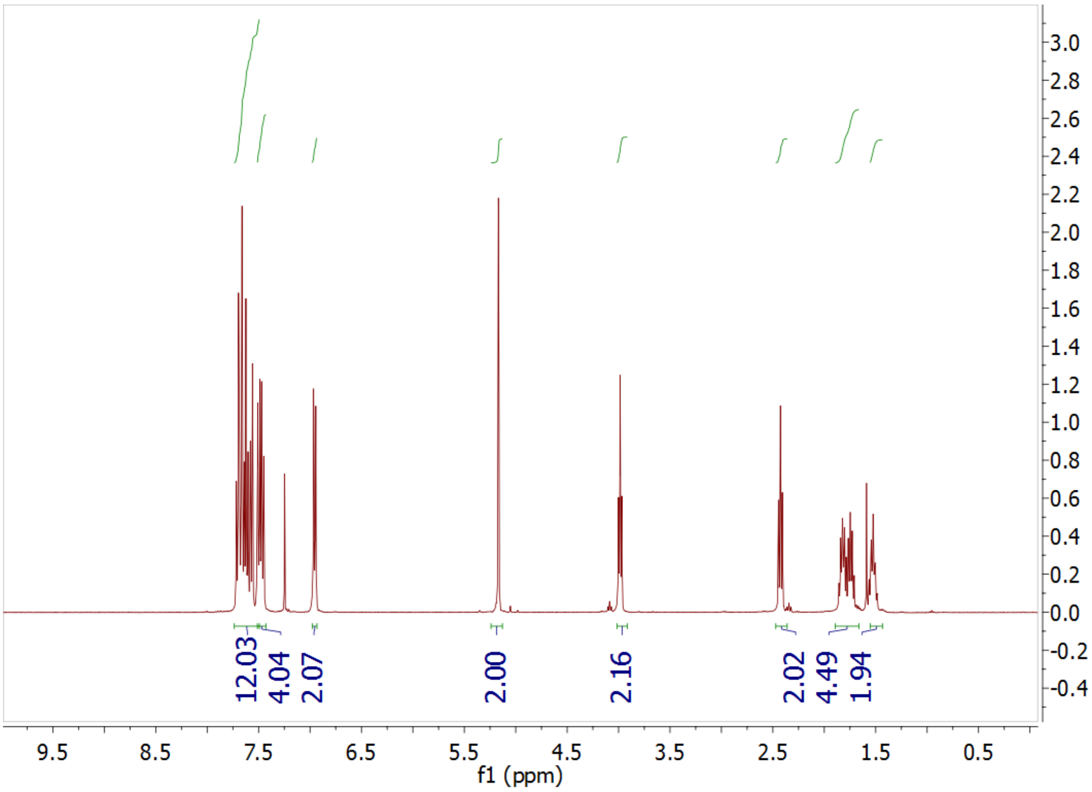

Figure SI-1: ^{1}H NMR (400 MHz, $CDCl_3$) of compound **9**

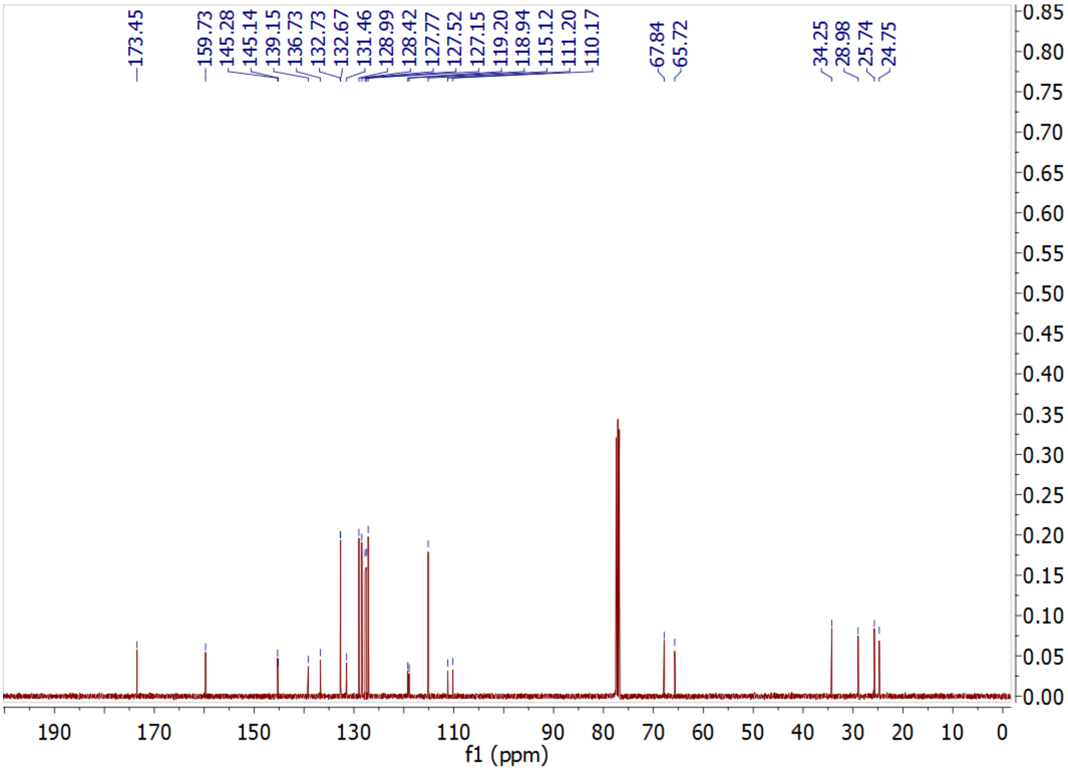

Figure SI-2: ^{13}C{^{1}H} NMR (100.5 MHz, $CDCl_3$) of compound **9**

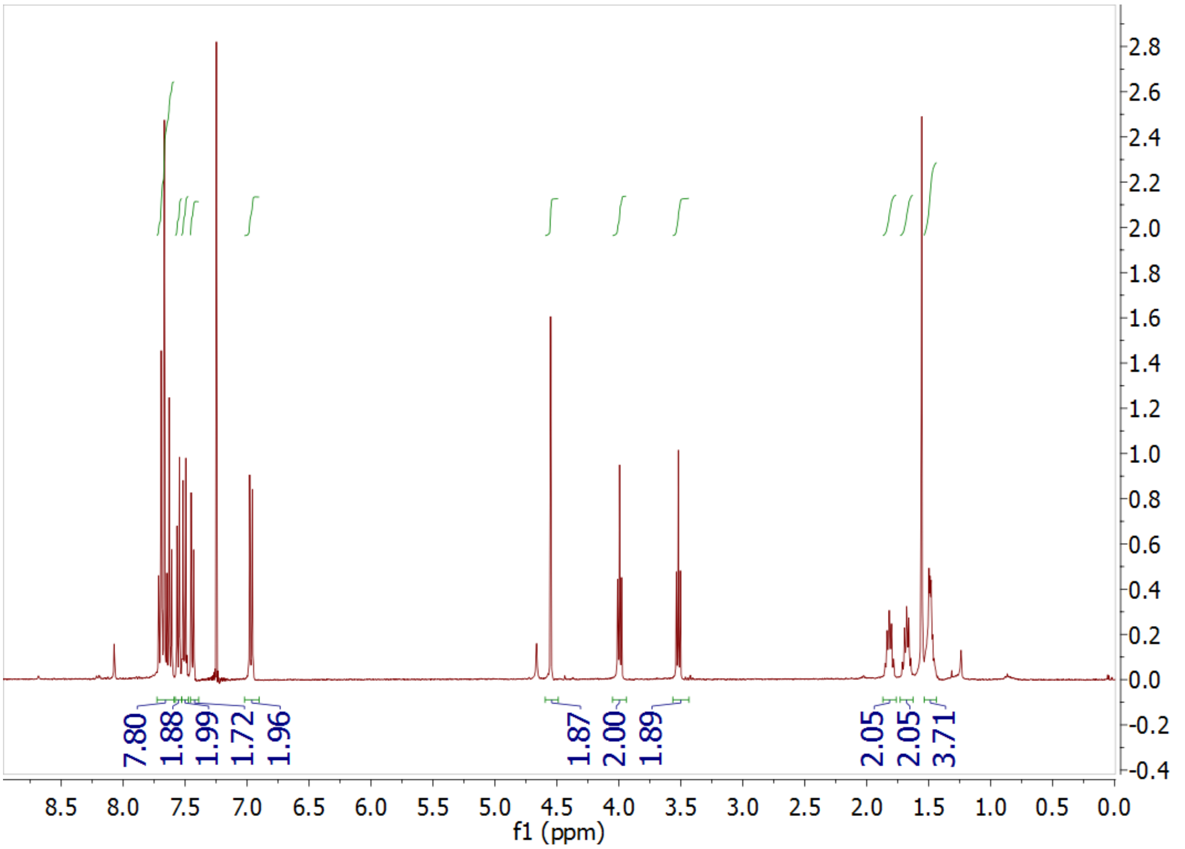

Figure SI-3: ^{1}H NMR (400 MHz, $CDCl_3$) of compound **10**

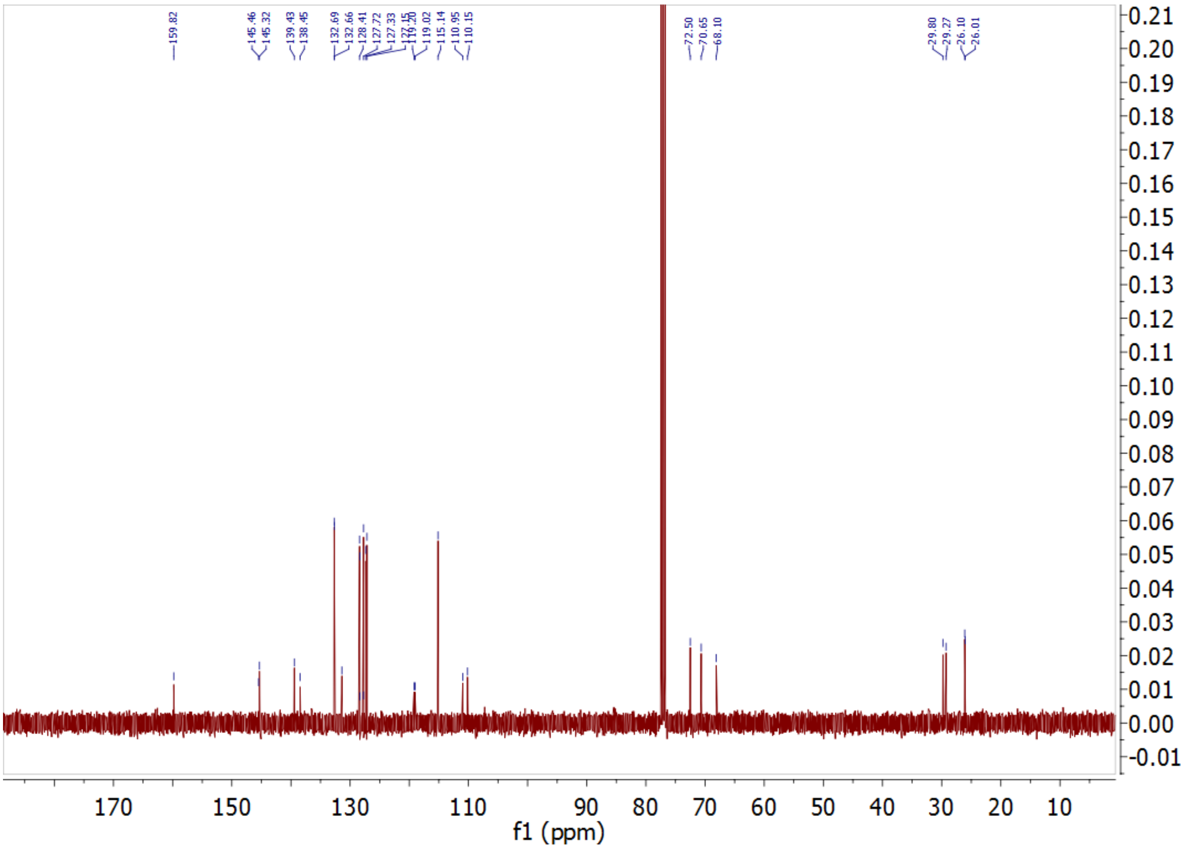

Figure SI-4: $^{13}C\{^{1}H\}$ NMR (100.5 MHz, $CDCl_3$) of compound **10**

4. References

1. H. M. Li, L. Q. Feng and X. H. Lou, *B Korean Chem Soc*, 2014, **35**, 2551-2554
2. Y. Xia, R. Verduzco, R. H. Grubbs and J. A. Kornfield, *J Am Chem Soc*, 2008, **130**, 1735-1740
3. E. J. Davis, R. J. Mandle, B. K. Russell, P. Y. Foeller, M. S. Cook, S. J. Cowling and J. W. Goodby, *Liq Cryst*, 2014, **41**, 1635-1646
4. M. J. Frisch, G. W. Trucks, H. B. Schlegel, G. E. Scuseria, M. A. Robb, J. R. Cheeseman, G. Scalmani, V. Barone, B. Mennucci, G. A. Petersson, H. Nakatsuji, M. Caricato, X. Li, H. P. Hratchian, A. F. Izmaylov, J. Bloino, G. Zheng, J. L. Sonnenberg, M. Hada, M. Ehara, K. Toyota, R. Fukuda, J. Hasegawa, M. Ishida, T. Nakajima, Y. Honda, O. Kitao, H. Nakai, T. Vreven, J. A. Montgomery Jr., J. E. Peralta, F. Ogliaro, M. J. Bearpark, J. Heyd, E. N. Brothers, K. N. Kudin, V. N. Staroverov, R. Kobayashi, J. Normand, K. Raghavachari, A. P. Rendell, J. C. Burant, S. S. Iyengar, J. Tomasi, M. Cossi, N. Rega, N. J. Millam, M. Klene, J. E. Knox, J. B. Cross, V. Bakken, C. Adamo, J. Jaramillo, R. Gomperts, R. E. Stratmann, O. Yazyev, A. J. Austin, R. Cammi, C. Pomelli, J. W. Ochterski, R. L. Martin, K. Morokuma, V. G. Zakrzewski, G. A. Voth, P. Salvador, J. J. Dannenberg, S. Dapprich, A. D. Daniels, Ö. Farkas, J. B. Foresman, J. V. Ortiz, J. Cioslowski and D. J. Fox, *Gaussian 09*, 2009
5. C. T. Archbold, R. J. Mandle, J. L. Andrews, S. J. Cowling and J. W. Goodby, *Liq Cryst*, 2017, **44**, 2079-2088
